# Supplementary material for: Characterization of Constitutional Ring Chromosomes over 37 Years of Experience at a Single-Site Institution
Source: Genes (Basel). 2025 Jun 25;16(7):736. doi: 10.3390/genes16070736 (PMC12295095; doi:10.3390/genes16070736)

Supplemental Table S1. The phenotype and laboratory testing details for individuals identified at GGC as having RCs.

Abbreviations: PB; peripheral blood, POC; products of conception, F; female, M; male, FTT; failure to thrive, DD; developmental delay, NOS; not otherwise specified, ID; intellectual disability, YO; year old, AD; autosomal dominant, SGA; small for gestational age, GA; gestational age, AFP; alpha-feto protein, NIPT; non-invasive prenatal testing/screening.

Supplemental Figures S1-S10. Available images for individuals identified at GGC as having RCs.

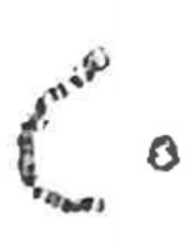

Supplemental Figure S1: RC4. A partial karyotype is depicted for RC4-1.

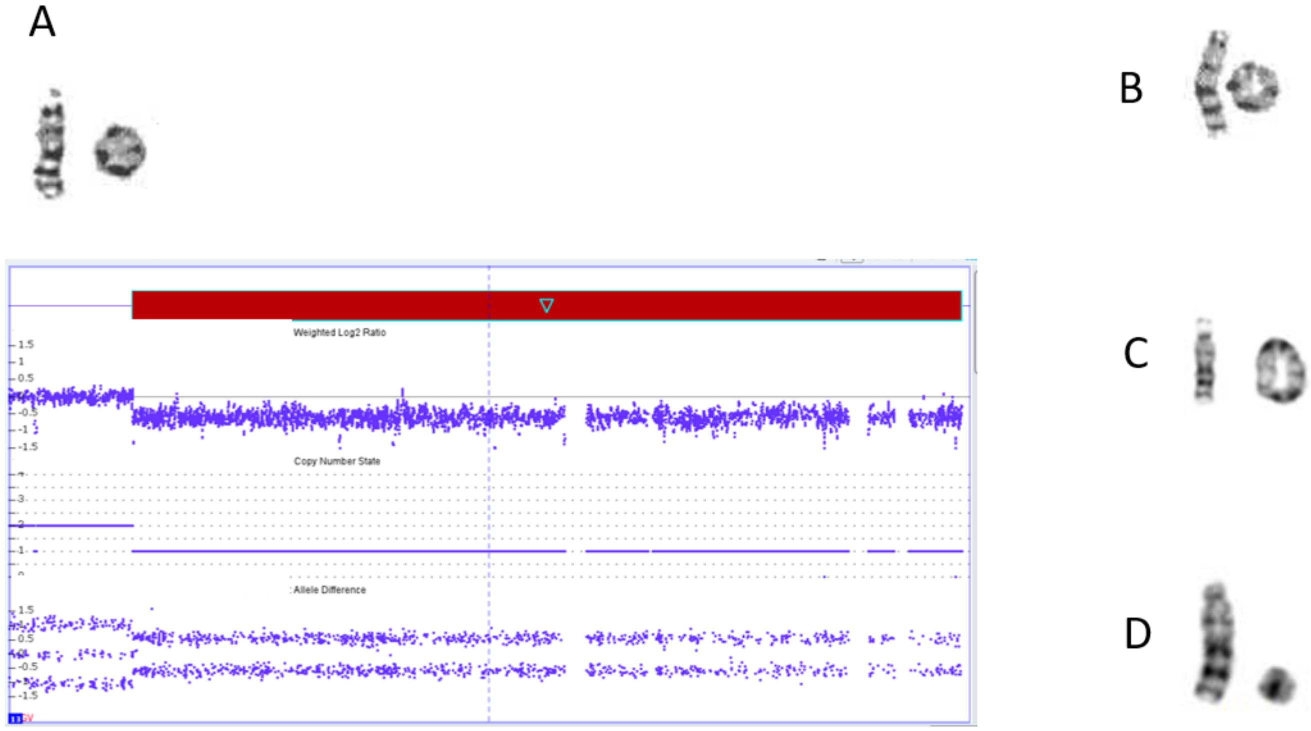

Supplemental Figure S2: RC13. Partial karyotypes are depicted for (RC13-1 (A), RC13-2 (B), RC13-3 (C), and RC13-4 (D)). The terminal 13q loss identified by CMA is also displayed at the chromosomal level (ChAS Browser Cytoscan HD Affymetrix (hg19)) for RC13-1 (A; lower panel).

Supplemental Figure S3: RC14. Partial karyotypes are depicted for (RC14-1 (A), RC14-2 (B), and RC14-3 (C). The terminal 14q loss is also displayed at the chromosomal level (Affymetrix Genome-wide Human SNP 6.0 Array (hg18)) for RC14-1 (A; lower) and (ChAS Browser Cytoscan HD Affymetrix hg19)) for RC14-3 (C; lower).

A

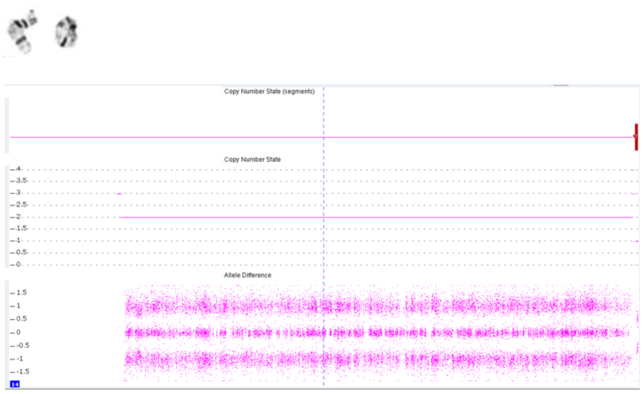

C

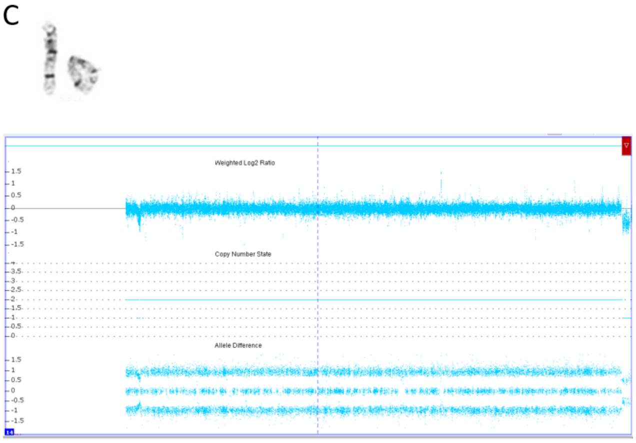

B

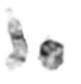

Supplemental Figure S4: RC15. A partial karyotype is depicted for RC15-1.

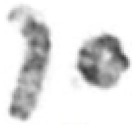

Supplemental Figure S5: RC18. Partial karyotypes are depicted for (RC18-1 (A), RC18-2 (B), RC18-3 (C), and RC18-4 (D)). The terminal 18p and 18q loss is also displayed at the chromosomal level (ChAS Browser Cytoscan HD Affymetrix hg19)) for RC18-4 (D; lower).

A

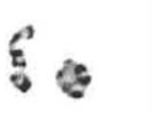

D

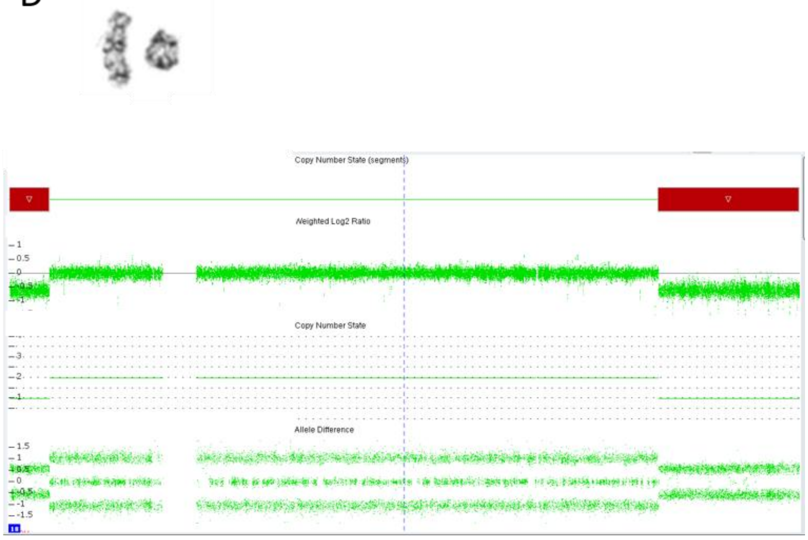

B

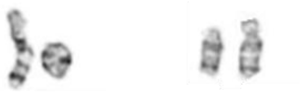

C

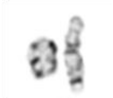

Supplemental Figure S6: RC21. Partial karyotypes are depicted for (RC21-1 (A), RC21-2 (B), RC21-3 (C), and RC21-4 (D)). The multiple copy number changes displayed at the chromosomal level (ChAS Browser CytoScan HD Affymetrix hg19)) for RC21-2 (B; lower) and RC21-3 (C; right). Images of historical CMA data using an OGT platform are available upon request for RC21-4.

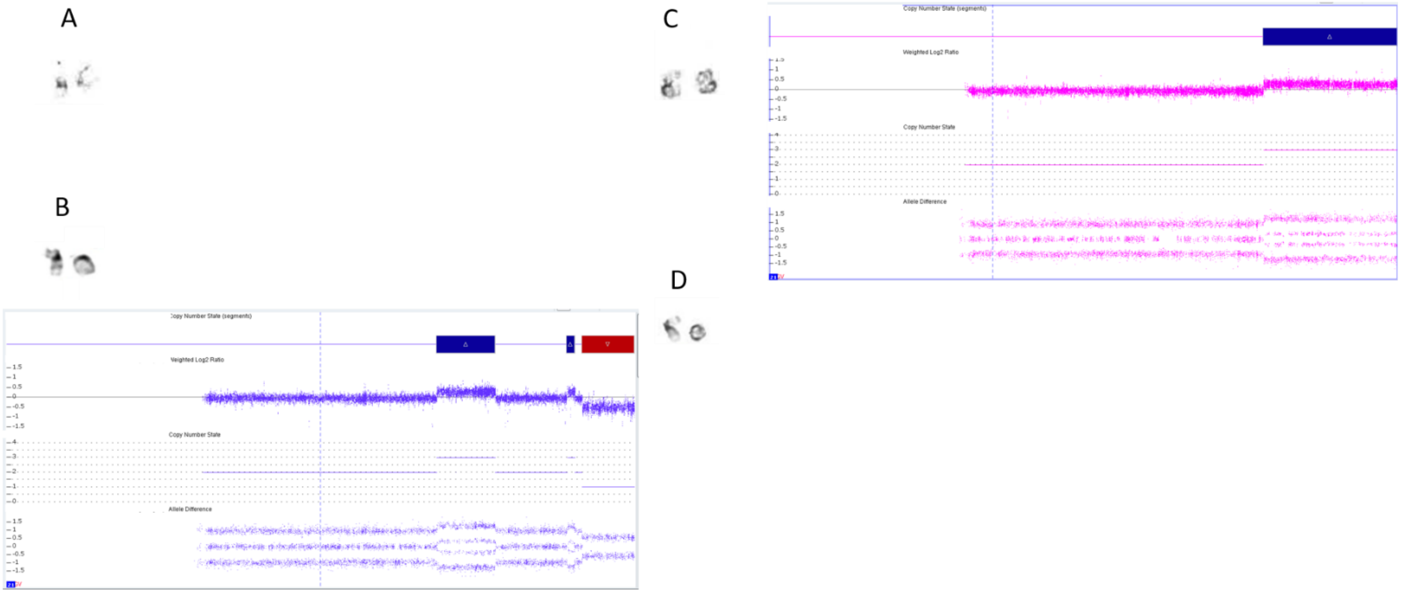

Supplemental Figure S7: RC22. Partial karyotypes are depicted for (RC22-1 (A), RC22-2 (B), RC22-3 (C), RC22-4 (D), RC22-5 (E), RC22-6 (F), RC22-7 (G), RC22-8 (H), RC22-9 including both inverted 9s (I), RC22-10 (J), RC22-11 (K), RC22-12 (L), RC22-13 (M), RC22-14 (N)). Representative FISH results confirm the deletion of chromosome 22qtel for RC22-12 (L; right). The terminal 22q loss is also displayed at the chromosomal level. (ChAS Browser CytoScan HD Affymetrix (hg19)) for RC22-6 (F; right). Images of historical CMA data using an OGT platform are available upon request for RC22-8, RC22-11, RC22-13 and RC22-14.

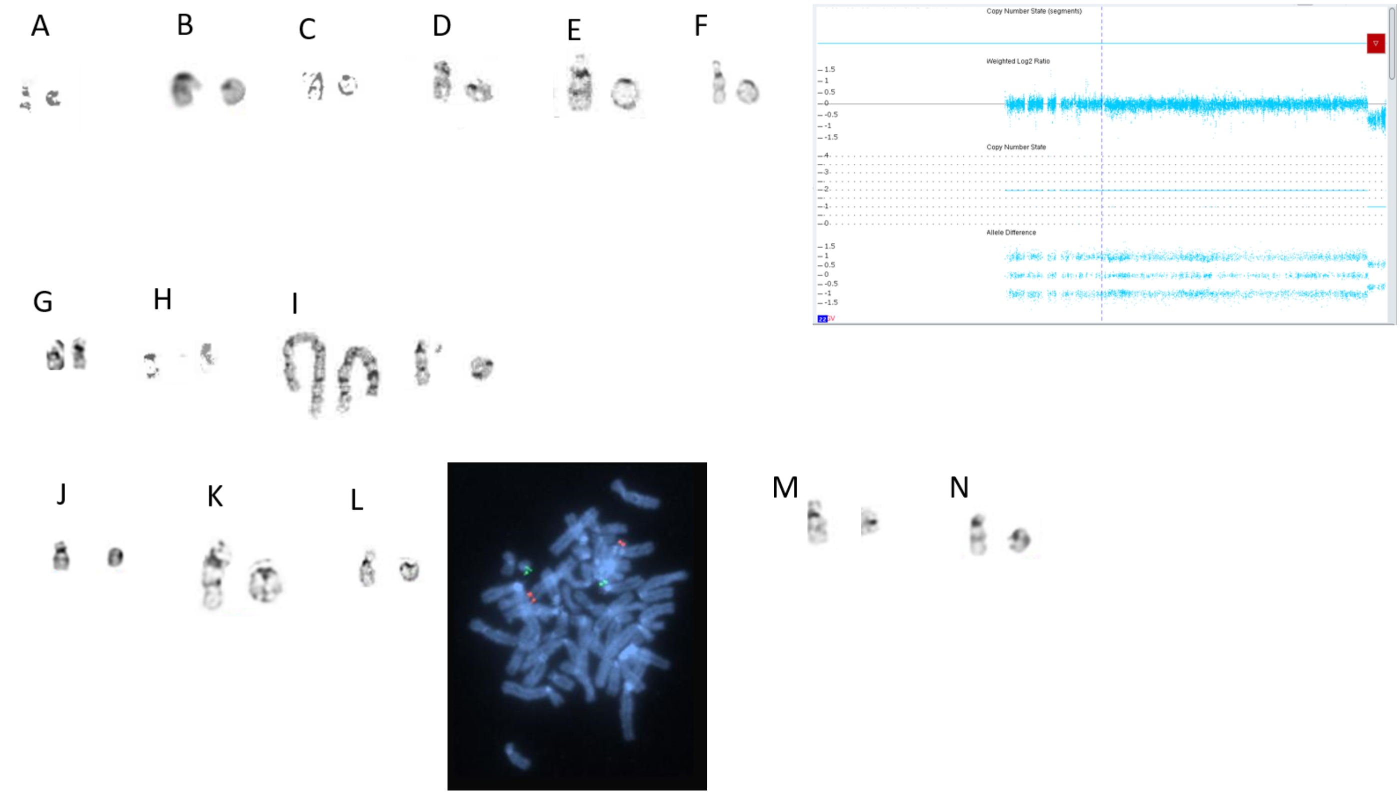

Supplemental Figure S8: RCX. Partial karyotypes are depicted for (RCX-1 (A), RCX-2 (B), RCX-3 (C), RCX-4 (D), RCX-5 (E), RCX-6 (F), and RCX-7 (G)). Representative FISH images demonstrate mosaicism with subsets with XX or monosomy X for RCX-3 and an

isochromosome 21q (C; lower). The X terminal arm losses and interstitial gain are also displayed at the chromosomal level. (ChAS Browser CytoScan HD Affymetrix (hg19)) for RCX-6 (F; lower).

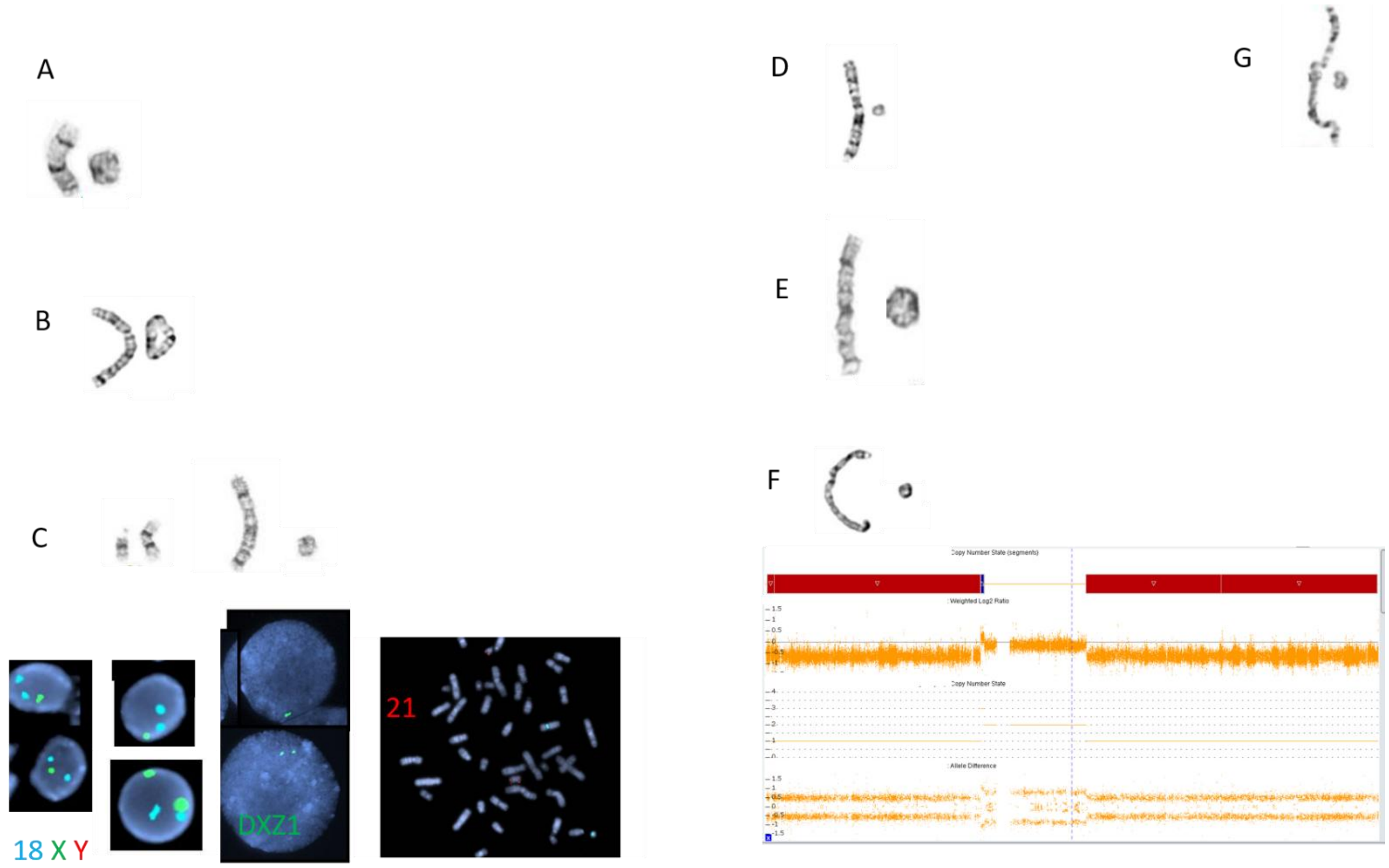

Supplemental Figure S9: RCY. Partial karyotypes are depicted for RCY-1. The X chromosome (right upper), for reference, and Y chromosome loss (right lower) are also displayed from the available CMA data (VIA Browser Bionano with GDA Cyto array (hg19)) for RCY-1. Here, the PAR1 region of X/Y and the PAR2 of X/Y demonstrate clinically significant copy number losses for Y, with an additional interstitial segmental loss of Yq identified.

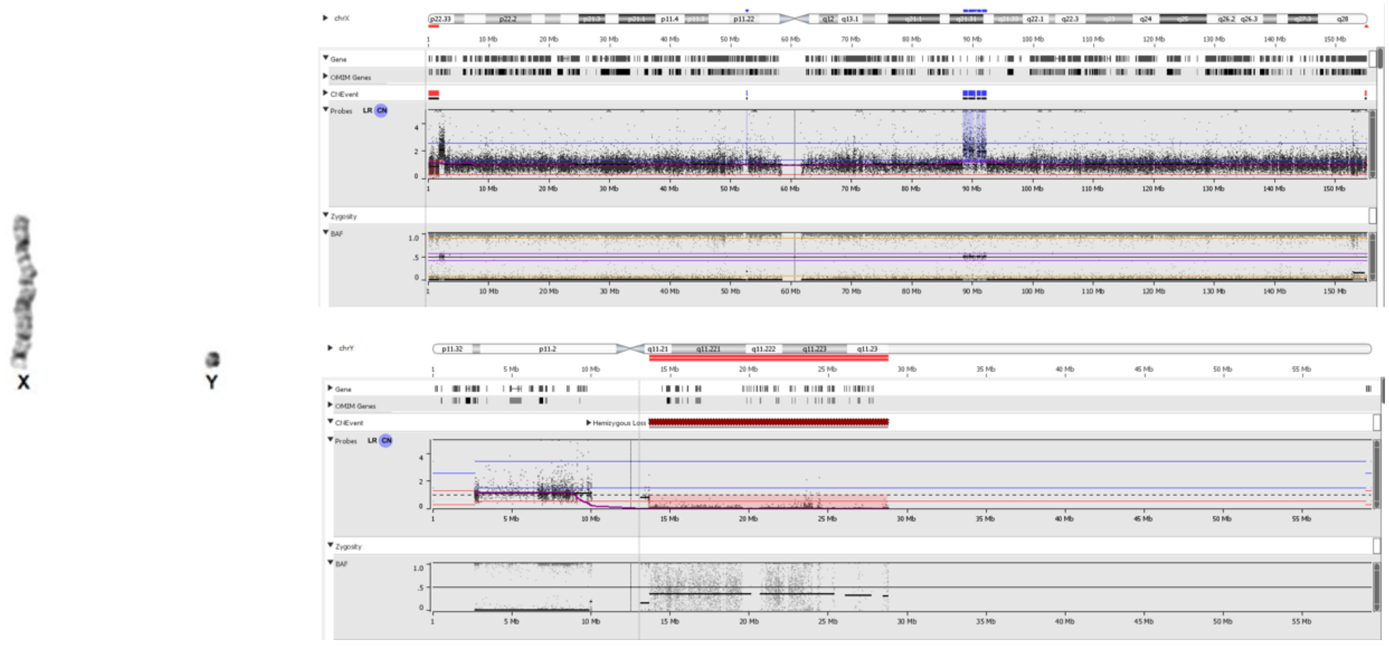

Supplemental Figure S10: RCX;Y. Partial X and Y chromosome karyotypes are depicted for RCX;Y-1 (far left). Representative FISH images (middle) demonstrate mosaicism for sex chromosome aneuploidy, with monosomy X and an apparently XY complement, and demonstrate Xp (STS) has localized to the RC. The Xp gain (right; upper) and terminal Yp and Yq loss (right; lower) identified by CMA are shown here (VIA Browser with GDA Cyto array (hg19)) for RCX;Y-1.

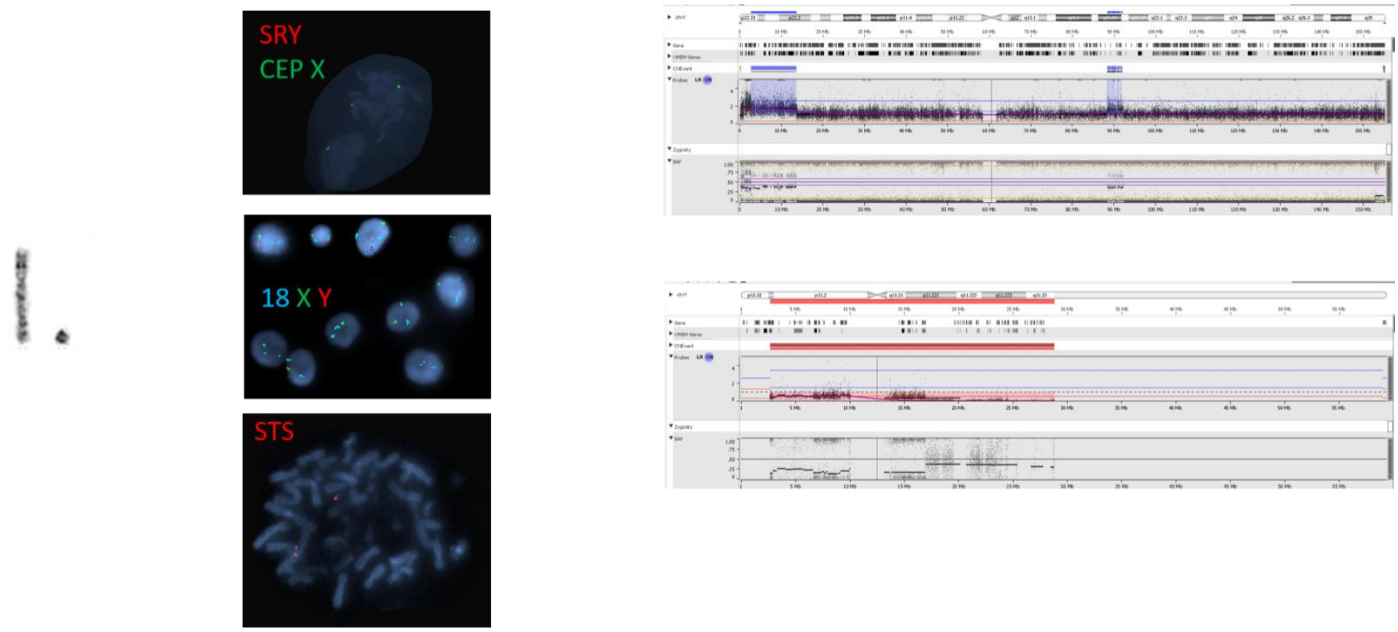

Supplement: Supplementary file 1 [file genes-16-00736-s001.zip › GGC_rings_060225_sup figs.pdf]
